# (2.2.2-Cryptand)potassium bis­(cyanato-κ*N*)(5,10,15,20-tetra­phenyl­por­phy­rin­ato-κ^4^ *N*)cobaltate(III) chloro­benzene hemisolvate

**DOI:** 10.1107/S1600536812038317

**Published:** 2012-09-15

**Authors:** Bader Belhaj Ali, Mohamed Salah Belkhiria, Jean-Claude Daran, Habib Nasri

**Affiliations:** a Université de Monastir, Faculté des Sciences de Monastir, Avenue de l’Environnement, 5019 Monastir, Tunisia; b Laboratoire de Chimie de Coordination, CNRS UPR 8241, 205 route de Narbonne, 31077 Toulouse, Cedex 04, France

## Abstract

In the title compound, [K(C_18_H_36_N_2_O_6_)][Co(NCO)_2_(C_44_H_28_N_4_)]·0.5C_6_H_5_Cl or [K(2,2,2-crypt)^+^][Co^III^(NCO)_2_(TPP)^−^]·0.5C_6_H_5_Cl, the Co^III^ ion is octa­hedrally coordin­ated by two axial N-bonded NCO^−^ anions and four pyrrole N atoms of the porphyrin. There is a major ruffling distortion of the porphyrin: the dihedral angles between *trans* pyrrole rings are 34.32 (14) and 34.72 (14)°. The potassium ion is coordinated by the six O atoms and two N atoms of the cryptand-222 mol­ecule and a weak K—O [3.407 (3) Å] bond to one of the cyanate O atoms also occurs. The packing also features weak C—H⋯O and C—H⋯π inter­actions. The contribution to the scattering of the disordered chloro­benzene solvent mol­ecules was removed with the SQUEEZE function in *PLATON* [Spek (2009 ▶). *Acta Cryst.* D**65**, 148–155].

## Related literature

For general background to cobalt and iron porphyrin species and their applications, see: Sanders *et al.* (2000 ▶); Dhifet *et al.* (2010 ▶); Mansour *et al.* (2010 ▶). For the synthesis of the [Co^II^(TPP)] complex, see: Madure & Scheidt (1976 ▶). For the synthesis of Co^II^ tetra­phenyl­porphyrins, see: Iimuna *et al.* (1988 ▶). For refinement details concerning the use of *SQUEEZE*, see: Spek (2009 ▶). For related structures, see: Englert *et al.* (2002 ▶); Bresciani-Pahor *et al.* (1990 ▶); Ali *et al.* (2011 ▶); Konarev *et al.* (2003 ▶). For a description of the Cambridge Structural Database, see: Allen (2002 ▶). For further details of geometric distortions in related compounds, see: Jentzen *et al.* (1997 ▶).
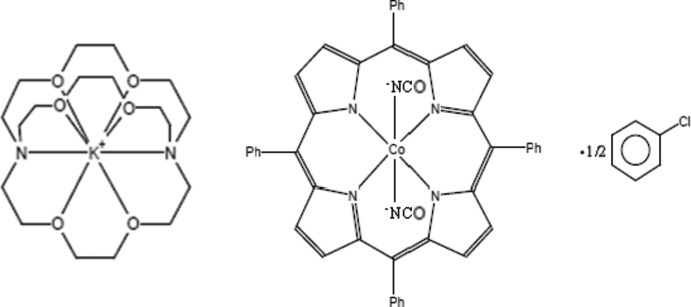


## Experimental

### 

#### Crystal data

[K(C_18_H_36_N_2_O_6_)][Co(NCO)_2_(C_44_H_28_N_4_)]·0.5C_6_H_5_Cl
*M* *_r_* = 1227.63
Monoclinic, 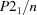

*a* = 14.7716 (5) Å
*b* = 23.7255 (9) Å
*c* = 18.0458 (7) Å
β = 90.325 (3)°
*V* = 6324.3 (4) Å^3^
*Z* = 4
Mo *K*α radiation
μ = 0.42 mm^−1^
*T* = 180 K
0.45 × 0.37 × 0.36 mm

#### Data collection

Agilent Xcalibur Sapphire2 diffractometer
Absorption correction: multi-scan (*CrysAlis PRO*; Agilent, 2010 ▶) *T*_min_ = 0.770, *T*_max_ = 1.000
32355 measured reflections
11118 independent reflections
8660 reflections with *I* > 2σ(*I*)
*R*_int_ = 0.041

#### Refinement

*R*[*F* ^2^ > 2σ(*F* ^2^)] = 0.045
*wR*(*F* ^2^) = 0.122
*S* = 1.08
11118 reflections
744 parameters
H-atom parameters constrained
Δρ_max_ = 0.48 e Å^−3^
Δρ_min_ = −0.44 e Å^−3^

### 

Data collection: *CrysAlis PRO* (Agilent, 2010 ▶); cell refinement: *CrysAlis PRO*; data reduction: *CrysAlis PRO*; program(s) used to solve structure: *SIR2004* (Burla *et al.*, 2005 ▶); program(s) used to refine structure: *SHELXL97* (Sheldrick, 2008 ▶); molecular graphics: *ORTEPIII* (Burnett & Johnson, 1996 ▶) and *ORTEP-3* (Farrugia, 1997 ▶); software used to prepare material for publication: *SHELXL97*.

## Supplementary Material

Crystal structure: contains datablock(s) I, New_Global_Publ_Block. DOI: 10.1107/S1600536812038317/hb6905sup1.cif

Structure factors: contains datablock(s) I. DOI: 10.1107/S1600536812038317/hb6905Isup2.hkl

Additional supplementary materials:  crystallographic information; 3D view; checkCIF report

## Figures and Tables

**Table 1:** Selected bond lengths (Å)

Co1—N6	1.905 (2)
Co1—N5	1.919 (2)
Co1—N1	1.9454 (19)
Co1—N4	1.947 (2)
Co1—N2	1.952 (2)
Co1—N3	1.9567 (19)

**Table 2:** Hydrogen-bond geometry (Å, °) *Cg*2 and *Cg*4 are the centroids of the N2/C6–C9 and N4/C16–C19 rings, respectively.

*D*—H⋯*A*	*D*—H	H⋯*A*	*D*⋯*A*	*D*—H⋯*A*
C50—H50*A*⋯O2^i^	0.97	2.59	3.555 (4)	171
C57—H57*B*⋯*Cg*2	0.97	2.83	3.783 (4)	168
C60—H60*B*⋯*Cg*4^ii^	0.97	2.60	3.437 (3)	145

Symmetry codes: (i) 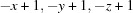; (ii) 
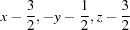
.

**Fig. 1.**
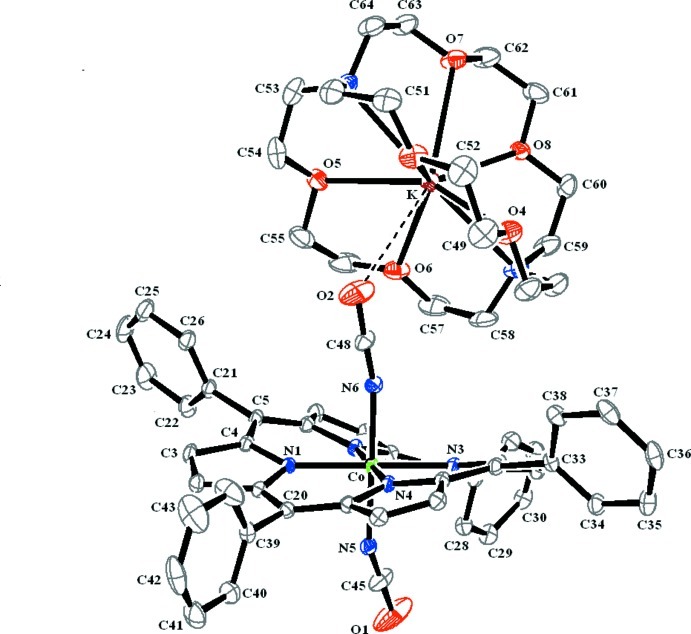
A view of the structure of (I). Displacement ellipsoids are drawn at 50% and H atoms have been omitted for clarity.

**Fig. 2.**
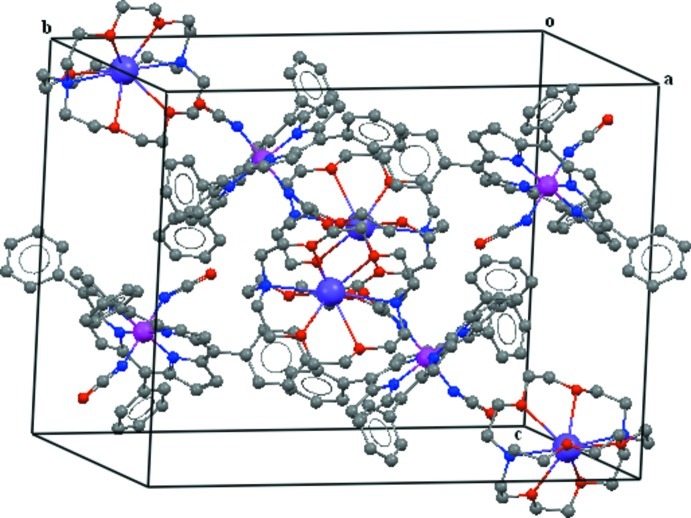
A unit-cell packing of (I). The H atoms have been omitted for clarity.

## supplementary crystallographic information

### Comment

Iron and cobalt porphyrin complexes have been used for many decades as biomimetic models for hemoproteines. These species are actually used in a variety of others domains (*i.e.*; catalysis, bio-sensors). Several iron and cobalt metalloporphyrins have been synthesized and characterized by our group (Dhifet *et al.*, 2010; Mansour *et al.*, 2010).

We report herein on the molecular structure of the title compound for which the asymmetric unit contains one ion complex [Co^III^(TPP)(NCO)_2_]^-^, the [K(2,2,2-crypt)]^+^ counterion and one half chlorobenzene solvent. For (I), the cobalt is coordinated to the four N atoms of the porphyrin ring and the N atoms from the two NCO^-^*trans* axial ligands (Fig.1). The two axial Co–N(NCO) bond lengths for (I) [1.905 (2) and 1.919 (2) Å] are in the range [1.898 (6) - 1.936 (7) Å] found for six-coordinated Co(III) complexes (CSD refcodes IFUVOU; Englert *et al.*, 2002 and KEYMEC; Bresciani-Pahor *et al.*, 1990) (CSD, version 5.32; Allen, 2002).

As seen in figure 1, the oxygen atom O2 of one cyanato-*N* axial ligand is weakly bonded to the potassium of the counterion [K(2,2,2-crypt)]^+^ with a distance of 3.407 (3) Å. The average K–O(2,2,2-crypt) distance is 2.831 (2) Å and the average K–N(2,2,2-crypt) bond length is 3.016 (2) Å.The porphyrin core is far from being planar, with deviations of atoms from the least-squares plane of CoN_4_C_20_, ranging from -0.586 (2) to 0.607 (2) Å. It is noteworthy the relationship between the ruffling of the porphyrin core and the mean equatorial Co—N_p_ distance; the CoN_4_C_20_ moiety is ruffled as the Co—N_p_ distance decreases, (Iimuna *et al.*, 1988). Thus, the practically planar porphyrin core of the ion complex [Co(OEP)(NO_2_)_2_]^-^ (OEP is the octaethylporphyrin) (Ali *et al.*, 2011) presents a Co—N_p_ of 1.988 (2) Å while for the very ruffled structure[Co^II^(TPP)] (Konarev *et al.*, 2003) the Co—N_p_ bond length value is 1.923 (4) Å. Therefore, the Co—N_p_ distance of (I) [1.950 (2) Å] is normal for a cobalt ruffled TPP complex. On the other hand Normal Structural Decomposition (NSD) calculations (Jentzen, *et al.*,1997) confirm the unusually important deformation of the porphyrin core with a major ruffling distortions of 78%.

The crystal packing features weak C—H······π interactions (Table 1 and Fig. 2)

### Experimental

The reaction of the [Co^II^(TPP)] complex (Madure & Scheidt, 1976) (15 mg, 0.022 mmol) with an excess of potasium cyanate KOCN (50 mg, 0.62 mmol) and cryptand-222 (50 mg, 0.13 mmol) in chlorobenzene (4 ml) under air overnight give a red-brown solution. Dark purple prisms of the title complex were obtained by diffusion of hexanes through the chlorobenzene solution.

### Refinement

Hydrogen atoms were placed using assumed geometrically idealized positions (C—H aromatic = 0.95 Å) and constrained to ride on their parent atoms, with U(H) = 1.2 *U*_eq_(C).

There are four cavities of 224 Å^3^ each. *PLATON* estimated that each cavity contains 33 electrons which may correspond to a half solvent molecule of chlorobenzene by asymmetric unit as suggested by chemical analyses. These residual electron density was difficult to modelize and therefore, the SQUEEZE function of *PLATON* (Spek, 2009) was used to eliminate the contribution of the electron density in the solvent region from the intensity data, and the solvent-free model was employed for the final refinement.

### Crystal data

**Table:**

[K(C_18_H_36_N_2_O_6_)][Co(NCO)_2_(C_44_H_28_N_4_)]·0.5C_6_H_5_Cl	*F*(000) = 2572
*M**_r_* = 1227.63	*D*_x_ = 1.289 Mg m^−^^3^
Monoclinic, *P*2_1_/*n*	Mo *K*α radiation, λ = 0.71073 Å
Hall symbol: -P 2yn	Cell parameters from 15544 reflections
*a* = 14.7716 (5) Å	θ = 2.9–28.5°
*b* = 23.7255 (9) Å	µ = 0.42 mm^−^^1^
*c* = 18.0458 (7) Å	*T* = 180 K
β = 90.325 (3)°	Prism, dark purple
*V* = 6324.3 (4) Å^3^	0.45 × 0.37 × 0.36 mm
*Z* = 4	

### Data collection

**Table:**

Agilent Xcalibur Sapphire2 diffractometer	11118 independent reflections
Radiation source: fine-focus sealed tube	8660 reflections with *I* > 2σ(*I*)
Graphite monochromator	*R*_int_ = 0.041
Detector resolution: 8.2632 pixels mm^-1^	θ_max_ = 25.4°, θ_min_ = 2.9°
ω scans	*h* = −17→17
Absorption correction: multi-scan (*CrysAlis PRO*; Agilent, 2010)	*k* = −28→28
*T*_min_ = 0.770, *T*_max_ = 1.000	*l* = −21→21
32355 measured reflections	

### Refinement

**Table:**

Refinement on *F*^2^	Primary atom site location: structure-invariant direct methods
Least-squares matrix: full	Secondary atom site location: difference Fourier map
*R*[*F*^2^ > 2σ(*F*^2^)] = 0.045	Hydrogen site location: inferred from neighbouring sites
*wR*(*F*^2^) = 0.122	H-atom parameters constrained
*S* = 1.08	*w* = 1/[σ^2^(*F*_o_^2^) + (0.059*P*)^2^ + 2.0072*P*] where *P* = (*F*_o_^2^ + 2*F*_c_^2^)/3
11118 reflections	(Δ/σ)_max_ = 0.001
744 parameters	Δρ_max_ = 0.48 e Å^−^^3^
0 restraints	Δρ_min_ = −0.44 e Å^−^^3^

### Special details

**Table:**

Geometry. All s.u.'s (except the s.u. in the dihedral angle between two l.s. planes) are estimated using the full covariance matrix. The cell s.u.'s are taken into account individually in the estimation of s.u.'s in distances, angles and torsion angles; correlations between s.u.'s in cell parameters are only used when they are defined by crystal symmetry. An approximate (isotropic) treatment of cell s.u.'s is used for estimating s.u.'s involving l.s. planes.
Refinement. Refinement of *F*\^2\^ against ALL reflections. The weighted *R*-factor *wR* and goodness of fit *S* are based on *F*\^2\^, conventional *R*-factors *R* are based on *F*, with *F* set to zero for negative *F*\^2\^. The threshold expression of *F*\^2\^ > σ(*F*\^2\^) is used only for calculating *R*-factors(gt) *etc*. and is not relevant to the choice of reflections for refinement. *R*-factors based on *F*\^2\^ are statistically about twice as large as those based on *F*, and *R*- factors based on ALL data will be even larger.

### Fractional atomic coordinates and isotropic or equivalent isotropic displacement parameters (Å^2^)

**Table:**

	*x*	*y*	*z*	*U*_iso_*/*U*_eq_	
Co1	0.41746 (2)	0.303450 (12)	0.765205 (17)	0.01882 (9)	
C1	0.47891 (17)	0.39649 (9)	0.86413 (13)	0.0235 (5)	
C2	0.44331 (18)	0.43899 (10)	0.91286 (14)	0.0276 (6)	
H2	0.4767	0.4632	0.9429	0.033*	
C3	0.35298 (19)	0.43709 (10)	0.90664 (14)	0.0294 (6)	
H3	0.3121	0.4605	0.9306	0.035*	
C4	0.33076 (17)	0.39238 (9)	0.85634 (13)	0.0230 (5)	
C5	0.24276 (17)	0.37482 (10)	0.84014 (13)	0.0253 (5)	
C6	0.22553 (17)	0.32218 (10)	0.80872 (13)	0.0244 (5)	
C7	0.13941 (18)	0.29475 (11)	0.80660 (14)	0.0299 (6)	
H7	0.0839	0.3110	0.8179	0.036*	
C8	0.15321 (18)	0.24125 (11)	0.78530 (14)	0.0293 (6)	
H8	0.1098	0.2129	0.7820	0.035*	
C9	0.24770 (17)	0.23612 (10)	0.76861 (13)	0.0238 (5)	
C10	0.28527 (17)	0.19042 (10)	0.73197 (13)	0.0241 (5)	
C11	0.36628 (17)	0.19416 (9)	0.69492 (13)	0.0224 (5)	
C12	0.39758 (18)	0.15473 (10)	0.64005 (14)	0.0278 (6)	
H12	0.3709	0.1204	0.6282	0.033*	
C13	0.47214 (18)	0.17655 (10)	0.60926 (14)	0.0270 (6)	
H13	0.5048	0.1612	0.5702	0.032*	
C14	0.49231 (17)	0.22821 (9)	0.64795 (13)	0.0223 (5)	
C15	0.57212 (17)	0.25833 (10)	0.64124 (13)	0.0235 (5)	
C16	0.59676 (17)	0.29995 (9)	0.69188 (13)	0.0223 (5)	
C17	0.68682 (17)	0.32199 (10)	0.69911 (14)	0.0264 (5)	
H17	0.7355	0.3143	0.6682	0.032*	
C18	0.68778 (17)	0.35588 (10)	0.75877 (13)	0.0264 (5)	
H18	0.7376	0.3752	0.7777	0.032*	
C19	0.59776 (16)	0.35666 (9)	0.78786 (13)	0.0225 (5)	
C20	0.56930 (17)	0.39076 (10)	0.84542 (13)	0.0238 (5)	
C21	0.16630 (17)	0.41203 (10)	0.86122 (14)	0.0281 (6)	
C22	0.10054 (19)	0.39637 (13)	0.91157 (16)	0.0397 (7)	
H22	0.1044	0.3617	0.9353	0.048*	
C23	0.0292 (2)	0.43226 (16)	0.92653 (19)	0.0533 (9)	
H23	−0.0150	0.4214	0.9601	0.064*	
C24	0.0229 (2)	0.48400 (15)	0.8922 (2)	0.0578 (10)	
H24	−0.0255	0.5078	0.9024	0.069*	
C25	0.0885 (2)	0.50028 (13)	0.84275 (19)	0.0481 (8)	
H25	0.0849	0.5353	0.8198	0.058*	
C26	0.15956 (19)	0.46445 (11)	0.82740 (16)	0.0346 (6)	
H26	0.2037	0.4756	0.7939	0.042*	
C27	0.23115 (17)	0.13731 (10)	0.72881 (14)	0.0249 (5)	
C28	0.22305 (19)	0.10503 (11)	0.79248 (15)	0.0319 (6)	
H28	0.2524	0.1162	0.8358	0.038*	
C29	0.17119 (19)	0.05600 (11)	0.79182 (16)	0.0360 (6)	
H29	0.1659	0.0346	0.8348	0.043*	
C30	0.12785 (19)	0.03908 (11)	0.72810 (16)	0.0372 (7)	
H30	0.0932	0.0064	0.7277	0.045*	
C31	0.1362 (2)	0.07110 (12)	0.66462 (17)	0.0407 (7)	
H31	0.1074	0.0597	0.6212	0.049*	
C32	0.1868 (2)	0.11986 (11)	0.66523 (15)	0.0353 (6)	
H32	0.1911	0.1413	0.6223	0.042*	
C33	0.63888 (17)	0.24296 (10)	0.58356 (13)	0.0254 (5)	
C34	0.68263 (19)	0.19139 (11)	0.58344 (14)	0.0332 (6)	
H34	0.6677	0.1645	0.6188	0.040*	
C35	0.7481 (2)	0.17922 (13)	0.53169 (16)	0.0416 (7)	
H35	0.7769	0.1444	0.5324	0.050*	
C36	0.7708 (2)	0.21855 (14)	0.47908 (16)	0.0420 (7)	
H36	0.8150	0.2104	0.4442	0.050*	
C37	0.7283 (2)	0.26982 (14)	0.47815 (15)	0.0417 (7)	
H37	0.7439	0.2965	0.4427	0.050*	
C38	0.66182 (18)	0.28206 (11)	0.52997 (14)	0.0306 (6)	
H38	0.6326	0.3168	0.5286	0.037*	
C39	0.63795 (17)	0.42845 (11)	0.88148 (14)	0.0284 (6)	
C40	0.6803 (2)	0.41314 (13)	0.94660 (16)	0.0444 (7)	
H40	0.6640	0.3799	0.9703	0.053*	
C41	0.7472 (2)	0.44727 (16)	0.9770 (2)	0.0573 (10)	
H41	0.7760	0.4363	1.0206	0.069*	
C42	0.7712 (2)	0.49646 (18)	0.9438 (2)	0.0615 (11)	
H42	0.8163	0.5190	0.9644	0.074*	
C43	0.7286 (3)	0.51238 (17)	0.8804 (2)	0.0758 (13)	
H43	0.7441	0.5462	0.8578	0.091*	
C44	0.6618 (3)	0.47817 (14)	0.84890 (19)	0.0600 (10)	
H44	0.6333	0.4894	0.8053	0.072*	
C45	0.4371 (2)	0.21365 (13)	0.87625 (18)	0.0491 (8)	
C46	0.38075 (19)	0.39021 (11)	0.64692 (15)	0.0335 (6)	
C47	0.3658 (2)	0.27299 (13)	0.4394 (2)	0.0548 (9)	
H47A	0.3628	0.2686	0.3860	0.066*	
H47B	0.3846	0.2371	0.4603	0.066*	
C48	0.4367 (2)	0.31679 (14)	0.4579 (2)	0.0562 (9)	
H48A	0.4383	0.3232	0.5110	0.067*	
H48B	0.4959	0.3035	0.4427	0.067*	
C49	0.4834 (2)	0.40924 (16)	0.4333 (2)	0.0605 (10)	
H49A	0.5414	0.3954	0.4163	0.073*	
H49B	0.4884	0.4170	0.4860	0.073*	
C50	0.4594 (2)	0.46173 (16)	0.3930 (2)	0.0612 (10)	
H50A	0.5095	0.4881	0.3954	0.073*	
H50B	0.4473	0.4532	0.3413	0.073*	
C51	0.3611 (2)	0.53924 (13)	0.39513 (19)	0.0509 (8)	
H51A	0.3442	0.5353	0.3434	0.061*	
H51B	0.4135	0.5638	0.3983	0.061*	
C52	0.2841 (2)	0.56410 (13)	0.43784 (19)	0.0514 (9)	
H52A	0.3003	0.5646	0.4900	0.062*	
H52B	0.2757	0.6029	0.4222	0.062*	
C53	0.1342 (3)	0.55264 (13)	0.4859 (2)	0.0576 (9)	
H53A	0.0732	0.5432	0.4699	0.069*	
H53B	0.1378	0.5933	0.4906	0.069*	
C54	0.1513 (3)	0.52678 (15)	0.5594 (2)	0.0592 (10)	
H54A	0.2147	0.5311	0.5726	0.071*	
H54B	0.1153	0.5457	0.5966	0.071*	
C55	0.1432 (3)	0.44287 (19)	0.62748 (18)	0.0674 (11)	
H55A	0.1043	0.4601	0.6642	0.081*	
H55B	0.2056	0.4482	0.6430	0.081*	
C56	0.1229 (3)	0.38185 (19)	0.6216 (2)	0.0713 (12)	
H56A	0.1225	0.3651	0.6706	0.086*	
H56B	0.0636	0.3765	0.5994	0.086*	
C57	0.1897 (3)	0.29637 (16)	0.5833 (2)	0.0693 (11)	
H57A	0.1350	0.2812	0.5610	0.083*	
H57B	0.1908	0.2856	0.6351	0.083*	
C58	0.2706 (3)	0.27272 (14)	0.54518 (19)	0.0585 (10)	
H58A	0.3246	0.2867	0.5698	0.070*	
H58B	0.2699	0.2320	0.5506	0.070*	
C59	0.2074 (2)	0.25531 (12)	0.4243 (2)	0.0550 (9)	
H59A	0.1514	0.2554	0.4520	0.066*	
H59B	0.2269	0.2164	0.4194	0.066*	
C60	0.1894 (2)	0.27852 (12)	0.34906 (18)	0.0494 (8)	
H60A	0.2447	0.2786	0.3204	0.059*	
H60B	0.1453	0.2552	0.3235	0.059*	
C61	0.1234 (3)	0.35410 (14)	0.28719 (18)	0.0569 (9)	
H61A	0.0830	0.3265	0.2652	0.068*	
H61B	0.1737	0.3599	0.2537	0.068*	
C62	0.0750 (3)	0.40724 (14)	0.2980 (2)	0.0630 (11)	
H62A	0.0468	0.4188	0.2517	0.076*	
H62B	0.0279	0.4022	0.3346	0.076*	
C63	0.0916 (2)	0.50148 (13)	0.3325 (2)	0.0552 (9)	
H63A	0.0459	0.4977	0.3706	0.066*	
H63B	0.0619	0.5128	0.2868	0.066*	
C64	0.1597 (2)	0.54490 (13)	0.35509 (19)	0.0535 (9)	
H64A	0.2082	0.5455	0.3191	0.064*	
H64B	0.1311	0.5817	0.3548	0.064*	
N1	0.40830 (13)	0.36760 (8)	0.83187 (10)	0.0214 (4)	
N4	0.54411 (13)	0.32126 (8)	0.74713 (10)	0.0212 (4)	
N3	0.42573 (13)	0.23860 (8)	0.69859 (10)	0.0210 (4)	
N2	0.29024 (14)	0.28625 (8)	0.78308 (11)	0.0227 (4)	
N5	0.45202 (15)	0.25569 (8)	0.84641 (11)	0.0264 (5)	
N6	0.38396 (15)	0.35109 (8)	0.68460 (11)	0.0274 (5)	
N7	0.27608 (18)	0.28639 (10)	0.46648 (14)	0.0430 (6)	
N8	0.19817 (17)	0.53412 (9)	0.42903 (14)	0.0410 (6)	
O5	0.12856 (15)	0.46853 (9)	0.55713 (11)	0.0465 (5)	
O6	0.18960 (15)	0.35528 (9)	0.57740 (11)	0.0495 (6)	
O7	0.13645 (15)	0.44939 (8)	0.32216 (12)	0.0486 (6)	
O8	0.15602 (14)	0.33395 (8)	0.35576 (10)	0.0404 (5)	
O3	0.38214 (14)	0.48576 (9)	0.42561 (12)	0.0486 (5)	
O4	0.41566 (14)	0.36779 (9)	0.42072 (13)	0.0508 (6)	
O1	0.4232 (2)	0.17042 (12)	0.9080 (2)	0.1138 (14)	
O2	0.3746 (2)	0.42972 (10)	0.60550 (16)	0.0767 (8)	
K1	0.24151 (4)	0.41042 (2)	0.45285 (3)	0.03328 (15)	

### Atomic displacement parameters (Å^2^)

**Table:**

	*U*^11^	*U*^22^	*U*^33^	*U*^12^	*U*^13^	*U*^23^
Co1	0.02012 (17)	0.01555 (15)	0.02083 (17)	0.00058 (13)	0.00273 (13)	−0.00084 (12)
C1	0.0275 (13)	0.0187 (11)	0.0244 (12)	0.0016 (10)	−0.0011 (10)	−0.0010 (9)
C2	0.0311 (14)	0.0226 (12)	0.0291 (14)	−0.0005 (11)	−0.0003 (11)	−0.0057 (10)
C3	0.0352 (15)	0.0224 (12)	0.0306 (14)	0.0055 (11)	0.0049 (12)	−0.0070 (10)
C4	0.0251 (13)	0.0195 (11)	0.0245 (12)	0.0025 (10)	0.0058 (10)	0.0001 (9)
C5	0.0271 (13)	0.0242 (12)	0.0246 (13)	0.0035 (11)	0.0068 (11)	−0.0013 (10)
C6	0.0243 (13)	0.0225 (12)	0.0263 (13)	0.0027 (10)	0.0040 (10)	0.0009 (10)
C7	0.0232 (13)	0.0336 (14)	0.0330 (14)	0.0010 (11)	0.0036 (11)	−0.0041 (11)
C8	0.0266 (14)	0.0299 (13)	0.0315 (14)	−0.0054 (11)	0.0051 (11)	−0.0041 (11)
C9	0.0239 (13)	0.0240 (12)	0.0235 (13)	−0.0045 (10)	0.0029 (10)	−0.0005 (10)
C10	0.0262 (13)	0.0203 (11)	0.0259 (13)	−0.0021 (10)	−0.0006 (11)	0.0011 (10)
C11	0.0267 (13)	0.0175 (11)	0.0230 (12)	−0.0014 (10)	0.0008 (10)	0.0016 (9)
C12	0.0316 (14)	0.0186 (11)	0.0331 (14)	−0.0030 (11)	−0.0005 (12)	−0.0054 (10)
C13	0.0305 (14)	0.0237 (12)	0.0269 (13)	0.0018 (11)	0.0045 (11)	−0.0067 (10)
C14	0.0255 (13)	0.0200 (11)	0.0215 (12)	0.0023 (10)	0.0048 (10)	−0.0003 (9)
C15	0.0251 (13)	0.0228 (12)	0.0225 (12)	0.0024 (10)	0.0035 (10)	0.0010 (9)
C16	0.0248 (13)	0.0195 (11)	0.0227 (12)	0.0004 (10)	0.0039 (10)	0.0021 (9)
C17	0.0233 (13)	0.0278 (12)	0.0280 (13)	0.0000 (11)	0.0072 (11)	0.0005 (10)
C18	0.0229 (13)	0.0268 (12)	0.0294 (13)	−0.0036 (11)	−0.0001 (11)	0.0000 (10)
C19	0.0240 (12)	0.0200 (11)	0.0237 (12)	−0.0006 (10)	0.0004 (10)	−0.0005 (9)
C20	0.0266 (13)	0.0210 (11)	0.0237 (12)	−0.0005 (10)	−0.0009 (10)	0.0021 (9)
C21	0.0242 (13)	0.0276 (13)	0.0324 (14)	0.0043 (11)	−0.0001 (11)	−0.0081 (11)
C22	0.0320 (16)	0.0477 (17)	0.0394 (16)	0.0060 (14)	0.0075 (13)	−0.0080 (13)
C23	0.0315 (17)	0.074 (2)	0.055 (2)	0.0108 (17)	0.0154 (15)	−0.0185 (18)
C24	0.0386 (19)	0.058 (2)	0.077 (2)	0.0253 (17)	−0.0063 (18)	−0.0314 (19)
C25	0.0456 (19)	0.0334 (15)	0.065 (2)	0.0136 (14)	−0.0168 (17)	−0.0156 (15)
C26	0.0335 (15)	0.0286 (13)	0.0418 (16)	0.0025 (12)	−0.0046 (13)	−0.0070 (11)
C27	0.0230 (13)	0.0183 (11)	0.0335 (14)	−0.0004 (10)	0.0058 (11)	−0.0010 (10)
C28	0.0334 (15)	0.0284 (13)	0.0340 (14)	−0.0059 (12)	0.0037 (12)	−0.0012 (11)
C29	0.0374 (16)	0.0299 (14)	0.0408 (16)	−0.0061 (12)	0.0104 (13)	0.0043 (12)
C30	0.0309 (15)	0.0254 (13)	0.0554 (18)	−0.0088 (12)	0.0091 (14)	−0.0043 (12)
C31	0.0465 (18)	0.0325 (14)	0.0432 (17)	−0.0109 (14)	−0.0078 (14)	−0.0055 (13)
C32	0.0424 (17)	0.0282 (13)	0.0354 (15)	−0.0066 (13)	−0.0007 (13)	0.0012 (11)
C33	0.0253 (13)	0.0293 (13)	0.0215 (12)	−0.0036 (11)	0.0039 (11)	−0.0048 (10)
C34	0.0359 (16)	0.0342 (14)	0.0296 (14)	0.0027 (12)	0.0092 (12)	−0.0026 (11)
C35	0.0348 (16)	0.0427 (16)	0.0475 (18)	0.0013 (14)	0.0121 (14)	−0.0158 (14)
C36	0.0332 (16)	0.0577 (19)	0.0355 (16)	−0.0083 (15)	0.0159 (13)	−0.0176 (14)
C37	0.0442 (18)	0.0562 (19)	0.0247 (14)	−0.0169 (16)	0.0079 (13)	−0.0007 (13)
C38	0.0302 (14)	0.0354 (14)	0.0262 (13)	−0.0076 (12)	0.0027 (11)	−0.0005 (11)
C39	0.0243 (13)	0.0303 (13)	0.0305 (14)	−0.0012 (11)	0.0026 (11)	−0.0106 (11)
C40	0.0481 (18)	0.0414 (16)	0.0435 (17)	0.0026 (15)	−0.0126 (15)	−0.0097 (13)
C41	0.047 (2)	0.070 (2)	0.055 (2)	0.0054 (19)	−0.0209 (17)	−0.0286 (18)
C42	0.0329 (17)	0.089 (3)	0.063 (2)	−0.0231 (19)	0.0139 (17)	−0.048 (2)
C43	0.092 (3)	0.069 (3)	0.066 (3)	−0.054 (2)	0.007 (2)	−0.011 (2)
C44	0.082 (3)	0.0515 (19)	0.0464 (19)	−0.0357 (19)	−0.0145 (18)	0.0040 (15)
C45	0.049 (2)	0.0412 (17)	0.057 (2)	0.0164 (15)	0.0276 (16)	0.0204 (16)
C46	0.0340 (16)	0.0243 (13)	0.0424 (16)	−0.0012 (12)	0.0089 (13)	0.0045 (12)
C47	0.053 (2)	0.0377 (17)	0.073 (2)	0.0085 (16)	−0.0169 (18)	−0.0033 (16)
C48	0.0392 (18)	0.056 (2)	0.074 (2)	0.0100 (16)	−0.0148 (17)	0.0026 (17)
C49	0.0287 (17)	0.070 (2)	0.083 (3)	−0.0081 (17)	0.0014 (17)	0.002 (2)
C50	0.043 (2)	0.064 (2)	0.076 (3)	−0.0132 (18)	0.0169 (19)	0.0054 (19)
C51	0.052 (2)	0.0425 (17)	0.058 (2)	−0.0203 (16)	−0.0025 (17)	0.0153 (15)
C52	0.062 (2)	0.0315 (15)	0.061 (2)	−0.0159 (16)	−0.0053 (18)	0.0033 (14)
C53	0.061 (2)	0.0330 (16)	0.079 (3)	0.0044 (16)	0.006 (2)	−0.0078 (16)
C54	0.059 (2)	0.061 (2)	0.058 (2)	−0.0080 (18)	0.0128 (18)	−0.0287 (18)
C55	0.062 (2)	0.107 (3)	0.0325 (18)	0.033 (2)	0.0034 (17)	0.0015 (19)
C56	0.060 (2)	0.099 (3)	0.055 (2)	0.017 (2)	0.0177 (19)	0.043 (2)
C57	0.084 (3)	0.065 (2)	0.059 (2)	−0.013 (2)	−0.002 (2)	0.0353 (19)
C58	0.074 (3)	0.0403 (17)	0.061 (2)	0.0016 (18)	−0.019 (2)	0.0234 (16)
C59	0.058 (2)	0.0271 (14)	0.079 (2)	−0.0070 (15)	−0.0261 (19)	0.0040 (15)
C60	0.051 (2)	0.0359 (16)	0.061 (2)	0.0028 (15)	−0.0174 (16)	−0.0191 (15)
C61	0.077 (3)	0.0516 (19)	0.0422 (19)	−0.0125 (18)	−0.0268 (17)	0.0014 (15)
C62	0.068 (2)	0.0461 (19)	0.075 (2)	−0.0117 (18)	−0.043 (2)	0.0085 (17)
C63	0.054 (2)	0.0421 (17)	0.069 (2)	0.0038 (16)	−0.0225 (17)	0.0076 (16)
C64	0.062 (2)	0.0330 (16)	0.065 (2)	−0.0026 (16)	−0.0159 (18)	0.0132 (15)
N1	0.0227 (10)	0.0195 (9)	0.0219 (10)	0.0020 (8)	0.0022 (8)	−0.0003 (8)
N4	0.0232 (11)	0.0192 (9)	0.0214 (10)	0.0016 (8)	0.0026 (8)	−0.0006 (8)
N3	0.0222 (11)	0.0189 (9)	0.0218 (10)	0.0000 (8)	0.0032 (9)	0.0008 (8)
N2	0.0239 (11)	0.0192 (9)	0.0250 (11)	−0.0001 (8)	0.0031 (9)	−0.0010 (8)
N5	0.0302 (12)	0.0249 (11)	0.0243 (11)	0.0026 (9)	0.0048 (9)	0.0009 (9)
N6	0.0302 (12)	0.0243 (11)	0.0275 (11)	−0.0001 (9)	−0.0003 (9)	0.0002 (9)
N7	0.0441 (15)	0.0316 (12)	0.0532 (16)	−0.0029 (11)	−0.0176 (12)	0.0067 (11)
N8	0.0438 (15)	0.0264 (11)	0.0527 (15)	−0.0045 (11)	−0.0012 (12)	0.0003 (10)
O5	0.0513 (13)	0.0543 (13)	0.0338 (11)	0.0044 (11)	−0.0017 (10)	−0.0026 (9)
O6	0.0465 (13)	0.0566 (13)	0.0453 (12)	0.0041 (11)	0.0040 (10)	0.0214 (10)
O7	0.0519 (13)	0.0363 (11)	0.0574 (13)	−0.0054 (10)	−0.0212 (11)	0.0077 (10)
O8	0.0535 (13)	0.0325 (10)	0.0350 (11)	−0.0011 (9)	−0.0174 (9)	−0.0040 (8)
O3	0.0372 (12)	0.0516 (13)	0.0571 (13)	−0.0105 (10)	0.0062 (10)	0.0151 (10)
O4	0.0354 (12)	0.0520 (13)	0.0649 (14)	−0.0023 (10)	−0.0073 (10)	0.0074 (11)
O1	0.115 (3)	0.0686 (19)	0.158 (3)	0.0402 (19)	0.081 (2)	0.078 (2)
O2	0.085 (2)	0.0508 (14)	0.095 (2)	−0.0036 (14)	0.0150 (16)	0.0461 (14)
K1	0.0364 (3)	0.0289 (3)	0.0346 (3)	−0.0047 (3)	0.0003 (3)	0.0018 (2)

### Geometric parameters (Å, º)

**Table:**

Co1—N6	1.905 (2)	C41—C42	1.359 (5)
Co1—N5	1.919 (2)	C41—H41	0.9300
Co1—N1	1.9454 (19)	C42—C43	1.356 (6)
Co1—N4	1.947 (2)	C42—H42	0.9300
Co1—N2	1.952 (2)	C43—C44	1.396 (5)
Co1—N3	1.9567 (19)	C43—H43	0.9300
C1—N1	1.375 (3)	C44—H44	0.9300
C1—C20	1.386 (3)	C45—N5	1.155 (3)
C1—C2	1.439 (3)	C45—O1	1.193 (4)
C2—C3	1.339 (4)	C46—N6	1.151 (3)
C2—H2	0.9300	C46—O2	1.202 (3)
C3—C4	1.433 (3)	C47—N7	1.450 (4)
C3—H3	0.9300	C47—C48	1.512 (5)
C4—N1	1.363 (3)	C47—H47A	0.9700
C4—C5	1.394 (3)	C47—H47B	0.9700
C5—C6	1.394 (3)	C48—O4	1.417 (4)
C5—C21	1.485 (3)	C48—H48A	0.9700
C6—N2	1.364 (3)	C48—H48B	0.9700
C6—C7	1.429 (4)	C49—O4	1.420 (4)
C7—C8	1.342 (4)	C49—C50	1.484 (5)
C7—H7	0.9300	C49—H49A	0.9700
C8—C9	1.435 (4)	C49—H49B	0.9700
C8—H8	0.9300	C50—O3	1.408 (4)
C9—N2	1.370 (3)	C50—H50A	0.9700
C9—C10	1.387 (3)	C50—H50B	0.9700
C10—C11	1.377 (4)	C51—O3	1.417 (4)
C10—C27	1.493 (3)	C51—C52	1.498 (5)
C11—N3	1.373 (3)	C51—H51A	0.9700
C11—C12	1.440 (3)	C51—H51B	0.9700
C12—C13	1.341 (4)	C52—N8	1.463 (4)
C12—H12	0.9300	C52—H52A	0.9700
C13—C14	1.441 (3)	C52—H52B	0.9700
C13—H13	0.9300	C53—N8	1.466 (4)
C14—N3	1.369 (3)	C53—C54	1.481 (5)
C14—C15	1.384 (3)	C53—H53A	0.9700
C15—C16	1.392 (3)	C53—H53B	0.9700
C15—C33	1.483 (3)	C54—O5	1.423 (4)
C16—N4	1.365 (3)	C54—H54A	0.9700
C16—C17	1.435 (3)	C54—H54B	0.9700
C17—C18	1.344 (3)	C55—O5	1.423 (4)
C17—H17	0.9300	C55—C56	1.482 (6)
C18—C19	1.432 (4)	C55—H55A	0.9700
C18—H18	0.9300	C55—H55B	0.9700
C19—N4	1.366 (3)	C56—O6	1.419 (4)
C19—C20	1.384 (3)	C56—H56A	0.9700
C20—C39	1.498 (3)	C56—H56B	0.9700
C21—C22	1.385 (4)	C57—O6	1.402 (4)
C21—C26	1.389 (4)	C57—C58	1.491 (5)
C22—C23	1.382 (4)	C57—H57A	0.9700
C22—H22	0.9300	C57—H57B	0.9700
C23—C24	1.378 (5)	C58—N7	1.460 (4)
C23—H23	0.9300	C58—H58A	0.9700
C24—C25	1.376 (5)	C58—H58B	0.9700
C24—H24	0.9300	C59—N7	1.464 (4)
C25—C26	1.380 (4)	C59—C60	1.487 (5)
C25—H25	0.9300	C59—H59A	0.9700
C26—H26	0.9300	C59—H59B	0.9700
C27—C32	1.382 (4)	C60—O8	1.410 (3)
C27—C28	1.387 (4)	C60—H60A	0.9700
C28—C29	1.393 (4)	C60—H60B	0.9700
C28—H28	0.9300	C61—O8	1.409 (3)
C29—C30	1.373 (4)	C61—C62	1.463 (5)
C29—H29	0.9300	C61—H61A	0.9700
C30—C31	1.381 (4)	C61—H61B	0.9700
C30—H30	0.9300	C62—O7	1.417 (4)
C31—C32	1.377 (4)	C62—H62A	0.9700
C31—H31	0.9300	C62—H62B	0.9700
C32—H32	0.9300	C63—O7	1.415 (4)
C33—C38	1.384 (4)	C63—C64	1.495 (5)
C33—C34	1.384 (4)	C63—H63A	0.9700
C34—C35	1.379 (4)	C63—H63B	0.9700
C34—H34	0.9300	C64—N8	1.470 (4)
C35—C36	1.374 (4)	C64—H64A	0.9700
C35—H35	0.9300	C64—H64B	0.9700
C36—C37	1.369 (4)	N7—K1	2.996 (2)
C36—H36	0.9300	N8—K1	3.034 (2)
C37—C38	1.390 (4)	O5—K1	2.874 (2)
C37—H37	0.9300	O6—K1	2.715 (2)
C38—H38	0.9300	O7—K1	2.964 (2)
C39—C44	1.365 (4)	O8—K1	2.8161 (18)
C39—C40	1.377 (4)	O3—K1	2.786 (2)
C40—C41	1.389 (4)	O4—K1	2.827 (2)
C40—H40	0.9300	O2—K1	3.407 (3)
			
N6—Co1—N5	179.60 (10)	C49—C50—H50A	109.9
N6—Co1—N1	89.38 (8)	O3—C50—H50B	109.9
N5—Co1—N1	90.51 (8)	C49—C50—H50B	109.9
N6—Co1—N4	89.37 (9)	H50A—C50—H50B	108.3
N5—Co1—N4	90.25 (8)	O3—C51—C52	108.6 (3)
N1—Co1—N4	90.23 (8)	O3—C51—H51A	110.0
N6—Co1—N2	90.23 (9)	C52—C51—H51A	110.0
N5—Co1—N2	90.15 (9)	O3—C51—H51B	110.0
N1—Co1—N2	89.48 (8)	C52—C51—H51B	110.0
N4—Co1—N2	179.51 (8)	H51A—C51—H51B	108.4
N6—Co1—N3	90.82 (8)	N8—C52—C51	114.4 (3)
N5—Co1—N3	89.29 (8)	N8—C52—H52A	108.7
N1—Co1—N3	179.48 (9)	C51—C52—H52A	108.7
N4—Co1—N3	90.25 (8)	N8—C52—H52B	108.7
N2—Co1—N3	90.04 (8)	C51—C52—H52B	108.7
N1—C1—C20	125.3 (2)	H52A—C52—H52B	107.6
N1—C1—C2	109.2 (2)	N8—C53—C54	113.2 (3)
C20—C1—C2	125.0 (2)	N8—C53—H53A	108.9
C3—C2—C1	107.0 (2)	C54—C53—H53A	108.9
C3—C2—H2	126.5	N8—C53—H53B	108.9
C1—C2—H2	126.5	C54—C53—H53B	108.9
C2—C3—C4	107.6 (2)	H53A—C53—H53B	107.7
C2—C3—H3	126.2	O5—C54—C53	109.7 (3)
C4—C3—H3	126.2	O5—C54—H54A	109.7
N1—C4—C5	126.0 (2)	C53—C54—H54A	109.7
N1—C4—C3	109.5 (2)	O5—C54—H54B	109.7
C5—C4—C3	124.3 (2)	C53—C54—H54B	109.7
C6—C5—C4	121.4 (2)	H54A—C54—H54B	108.2
C6—C5—C21	120.0 (2)	O5—C55—C56	108.9 (3)
C4—C5—C21	118.6 (2)	O5—C55—H55A	109.9
N2—C6—C5	124.8 (2)	C56—C55—H55A	109.9
N2—C6—C7	109.4 (2)	O5—C55—H55B	109.9
C5—C6—C7	125.4 (2)	C56—C55—H55B	109.9
C8—C7—C6	107.6 (2)	H55A—C55—H55B	108.3
C8—C7—H7	126.2	O6—C56—C55	109.5 (3)
C6—C7—H7	126.2	O6—C56—H56A	109.8
C7—C8—C9	106.8 (2)	C55—C56—H56A	109.8
C7—C8—H8	126.6	O6—C56—H56B	109.8
C9—C8—H8	126.6	C55—C56—H56B	109.8
N2—C9—C10	125.8 (2)	H56A—C56—H56B	108.2
N2—C9—C8	109.4 (2)	O6—C57—C58	109.9 (3)
C10—C9—C8	123.9 (2)	O6—C57—H57A	109.7
C11—C10—C9	122.1 (2)	C58—C57—H57A	109.7
C11—C10—C27	120.2 (2)	O6—C57—H57B	109.7
C9—C10—C27	117.5 (2)	C58—C57—H57B	109.7
N3—C11—C10	125.7 (2)	H57A—C57—H57B	108.2
N3—C11—C12	108.9 (2)	N7—C58—C57	114.5 (3)
C10—C11—C12	125.1 (2)	N7—C58—H58A	108.6
C13—C12—C11	107.6 (2)	C57—C58—H58A	108.6
C13—C12—H12	126.2	N7—C58—H58B	108.6
C11—C12—H12	126.2	C57—C58—H58B	108.6
C12—C13—C14	107.2 (2)	H58A—C58—H58B	107.6
C12—C13—H13	126.4	N7—C59—C60	114.1 (3)
C14—C13—H13	126.4	N7—C59—H59A	108.7
N3—C14—C15	125.5 (2)	C60—C59—H59A	108.7
N3—C14—C13	109.2 (2)	N7—C59—H59B	108.7
C15—C14—C13	124.8 (2)	C60—C59—H59B	108.7
C14—C15—C16	121.9 (2)	H59A—C59—H59B	107.6
C14—C15—C33	120.3 (2)	O8—C60—C59	109.2 (2)
C16—C15—C33	117.5 (2)	O8—C60—H60A	109.9
N4—C16—C15	126.5 (2)	C59—C60—H60A	109.9
N4—C16—C17	109.3 (2)	O8—C60—H60B	109.9
C15—C16—C17	123.8 (2)	C59—C60—H60B	109.9
C18—C17—C16	107.2 (2)	H60A—C60—H60B	108.3
C18—C17—H17	126.4	O8—C61—C62	109.9 (3)
C16—C17—H17	126.4	O8—C61—H61A	109.7
C17—C18—C19	107.2 (2)	C62—C61—H61A	109.7
C17—C18—H18	126.4	O8—C61—H61B	109.7
C19—C18—H18	126.4	C62—C61—H61B	109.7
N4—C19—C20	125.8 (2)	H61A—C61—H61B	108.2
N4—C19—C18	109.4 (2)	O7—C62—C61	109.7 (3)
C20—C19—C18	124.7 (2)	O7—C62—H62A	109.7
C19—C20—C1	122.5 (2)	C61—C62—H62A	109.7
C19—C20—C39	117.9 (2)	O7—C62—H62B	109.7
C1—C20—C39	119.1 (2)	C61—C62—H62B	109.7
C22—C21—C26	118.7 (3)	H62A—C62—H62B	108.2
C22—C21—C5	123.1 (2)	O7—C63—C64	108.8 (3)
C26—C21—C5	118.2 (2)	O7—C63—H63A	109.9
C23—C22—C21	120.1 (3)	C64—C63—H63A	109.9
C23—C22—H22	120.0	O7—C63—H63B	109.9
C21—C22—H22	120.0	C64—C63—H63B	109.9
C24—C23—C22	120.7 (3)	H63A—C63—H63B	108.3
C24—C23—H23	119.7	N8—C64—C63	112.6 (3)
C22—C23—H23	119.7	N8—C64—H64A	109.1
C25—C24—C23	119.7 (3)	C63—C64—H64A	109.1
C25—C24—H24	120.2	N8—C64—H64B	109.1
C23—C24—H24	120.2	C63—C64—H64B	109.1
C24—C25—C26	119.8 (3)	H64A—C64—H64B	107.8
C24—C25—H25	120.1	C4—N1—C1	106.55 (19)
C26—C25—H25	120.1	C4—N1—Co1	126.81 (16)
C25—C26—C21	121.1 (3)	C1—N1—Co1	126.64 (16)
C25—C26—H26	119.5	C16—N4—C19	106.8 (2)
C21—C26—H26	119.5	C16—N4—Co1	126.43 (16)
C32—C27—C28	118.7 (2)	C19—N4—Co1	126.74 (16)
C32—C27—C10	122.4 (2)	C14—N3—C11	106.93 (19)
C28—C27—C10	118.9 (2)	C14—N3—Co1	126.82 (16)
C27—C28—C29	120.3 (2)	C11—N3—Co1	126.24 (16)
C27—C28—H28	119.9	C6—N2—C9	106.6 (2)
C29—C28—H28	119.9	C6—N2—Co1	127.08 (16)
C30—C29—C28	120.3 (3)	C9—N2—Co1	126.24 (17)
C30—C29—H29	119.8	C45—N5—Co1	144.6 (2)
C28—C29—H29	119.8	C46—N6—Co1	159.8 (2)
C29—C30—C31	119.4 (2)	C47—N7—C58	109.6 (3)
C29—C30—H30	120.3	C47—N7—C59	110.2 (3)
C31—C30—H30	120.3	C58—N7—C59	110.6 (3)
C32—C31—C30	120.4 (3)	C47—N7—K1	110.08 (18)
C32—C31—H31	119.8	C58—N7—K1	106.72 (19)
C30—C31—H31	119.8	C59—N7—K1	109.55 (17)
C31—C32—C27	120.9 (3)	C52—N8—C53	109.9 (2)
C31—C32—H32	119.6	C52—N8—C64	110.2 (2)
C27—C32—H32	119.6	C53—N8—C64	109.6 (3)
C38—C33—C34	118.4 (2)	C52—N8—K1	105.81 (18)
C38—C33—C15	119.5 (2)	C53—N8—K1	109.09 (17)
C34—C33—C15	122.1 (2)	C64—N8—K1	112.16 (17)
C35—C34—C33	121.0 (3)	C54—O5—C55	110.8 (3)
C35—C34—H34	119.5	C54—O5—K1	110.26 (18)
C33—C34—H34	119.5	C55—O5—K1	107.03 (19)
C36—C35—C34	120.1 (3)	C57—O6—C56	113.6 (3)
C36—C35—H35	120.0	C57—O6—K1	122.9 (2)
C34—C35—H35	120.0	C56—O6—K1	116.86 (18)
C37—C36—C35	119.8 (3)	C63—O7—C62	110.9 (3)
C37—C36—H36	120.1	C63—O7—K1	114.28 (18)
C35—C36—H36	120.1	C62—O7—K1	110.89 (17)
C36—C37—C38	120.2 (3)	C61—O8—C60	111.0 (2)
C36—C37—H37	119.9	C61—O8—K1	118.53 (17)
C38—C37—H37	119.9	C60—O8—K1	119.91 (16)
C33—C38—C37	120.5 (3)	C50—O3—C51	112.2 (2)
C33—C38—H38	119.8	C50—O3—K1	114.90 (19)
C37—C38—H38	119.8	C51—O3—K1	118.75 (18)
C44—C39—C40	118.6 (3)	C48—O4—C49	111.3 (2)
C44—C39—C20	120.3 (2)	C48—O4—K1	113.93 (19)
C40—C39—C20	121.1 (2)	C49—O4—K1	111.12 (19)
C39—C40—C41	120.1 (3)	C46—O2—K1	116.0 (2)
C39—C40—H40	119.9	O6—K1—O3	132.03 (7)
C41—C40—H40	119.9	O6—K1—O8	94.41 (7)
C42—C41—C40	120.9 (3)	O3—K1—O8	129.47 (7)
C42—C41—H41	119.6	O6—K1—O4	105.01 (7)
C40—C41—H41	119.6	O3—K1—O4	60.87 (7)
C43—C42—C41	119.4 (3)	O8—K1—O4	92.71 (6)
C43—C42—H42	120.3	O6—K1—O5	61.40 (7)
C41—C42—H42	120.3	O3—K1—O5	104.12 (7)
C42—C43—C44	120.3 (4)	O8—K1—O5	117.15 (7)
C42—C43—H43	119.8	O4—K1—O5	146.94 (6)
C44—C43—H43	119.8	O6—K1—O7	131.30 (7)
C39—C44—C43	120.7 (3)	O3—K1—O7	92.70 (6)
C39—C44—H44	119.6	O8—K1—O7	58.33 (5)
C43—C44—H44	119.6	O4—K1—O7	114.92 (7)
N5—C45—O1	178.7 (5)	O5—K1—O7	93.90 (6)
N6—C46—O2	177.0 (3)	O6—K1—N7	60.51 (7)
N7—C47—C48	114.1 (3)	O3—K1—N7	121.17 (7)
N7—C47—H47A	108.7	O8—K1—N7	59.61 (6)
C48—C47—H47A	108.7	O4—K1—N7	60.71 (7)
N7—C47—H47B	108.7	O5—K1—N7	121.10 (7)
C48—C47—H47B	108.7	O7—K1—N7	117.38 (6)
H47A—C47—H47B	107.6	O6—K1—N8	121.53 (7)
O4—C48—C47	109.4 (3)	O3—K1—N8	60.75 (7)
O4—C48—H48A	109.8	O8—K1—N8	116.20 (6)
C47—C48—H48A	109.8	O4—K1—N8	120.57 (7)
O4—C48—H48B	109.8	O5—K1—N8	60.39 (7)
C47—C48—H48B	109.8	O7—K1—N8	58.42 (6)
H48A—C48—H48B	108.2	N7—K1—N8	175.79 (7)
O4—C49—C50	109.7 (3)	O6—K1—O2	63.88 (6)
O4—C49—H49A	109.7	O3—K1—O2	68.21 (6)
C50—C49—H49A	109.7	O8—K1—O2	147.62 (6)
O4—C49—H49B	109.7	O4—K1—O2	72.07 (7)
C50—C49—H49B	109.7	O5—K1—O2	74.97 (7)
H49A—C49—H49B	108.2	O7—K1—O2	154.01 (6)
O3—C50—C49	109.1 (3)	N7—K1—O2	88.18 (6)
O3—C50—H50A	109.9	N8—K1—O2	96.02 (6)

### Hydrogen-bond geometry (Å, º)

Cg2 and Cg4 are the centroids of the N2/C6–C9 and N4/C16–C19 rings, respectively.

**Table:**

*D*—H···*A*	*D*—H	H···*A*	*D*···*A*	*D*—H···*A*
C50—H50*A*···O2^i^	0.97	2.59	3.555 (4)	171
C57—H57*B*···*Cg*2	0.97	2.83	3.783 (4)	168
C60—H60*B*···*Cg*4^ii^	0.97	2.60	3.437 (3)	145

Symmetry codes: (i) −*x*+1, −*y*+1, −*z*+1; (ii) *x*−3/2, −*y*−1/2, *z*−3/2.
